# Emerging immunotherapeutic strategies for malignant serous effusions: current evidence and future directions

**DOI:** 10.3389/fonc.2026.1757825

**Published:** 2026-01-21

**Authors:** Weimin He, Xia He, Zhi Qiao, Yongsheng Wang

**Affiliations:** 1 Division of Thoracic Tumor Multimodality Treatment, Cancer Center, West China Hospital, Sichuan University, Chengdu, China; 2 Clinical Trial Center, National Medical Products Administration Key Laboratory for Clinical Research and Evaluation of Innovative Drugs, West China Hospital, Sichuan University, Chengdu, China; 3 Division of Abdominal Tumor Multimodality Treatment, Cancer Center, West China Hospital, Sichuan University, Chengdu, China

**Keywords:** chimeric antigen receptor T cells (CAR-T), immune escape, immunotherapy, malignant serous effusion, tumor microenvironment

## Abstract

The invasion of serous cavities by malignant tumors, leading to implantation metastasis, is a hallmark of advanced cancer, causing pathological buildup of fluid (malignant effusions) containing cancerous cells, which indicates advanced disease and poor prognosis. The primary cancers involved include lung, breast, gastric, and ovarian cancers, affecting a large patient population with a high incidence rate. Because effective treatment options are limited in clinical practice, patients with malignant effusion, based on the severity, typically survive only 3 to 15 months. Traditional therapies, such as local drainage combined with intrapleural chemotherapy, have limited efficacy and tolerability. Immunotherapy has emerged as a potential approach for drug-resistant malignant tumors, driven by deeper insights into resistance mechanisms, tumor heterogeneity, and the immune microenvironment of malignant effusions. Preclinical studies show that liposomal nanoparticles containing cyclic dinucleotides (LNP-CDN) can activate the STING (Stimulator of Interferon Genes) signaling pathway; local injection into mouse pleural cavities enhances the “cold” tumor immune response and improves tumor treatment outcomes. In early-phase clinical studies and small cohorts, local infusion of chimeric antigen receptor T cells (CAR-T) showed preliminary survival benefits over first-line standard treatments, with good safety profiles and high tolerable doses, but these findings require further validation in large-scale trials. This article reviews tumor serosal metastasis epidemiology, summarizes current treatment options, and discusses the development direction of clinical strategies considering the immune microenvironment of malignant effusions, with a focus on advancements in cell immunotherapy. Despite the therapeutic potential of immunotherapy, its efficacy in malignant effusions, while measurable, remains to be fully optimized. A key limitation is that most evidence presented derives from preclinical models, small patient cohorts, or phase I/II trials, and definitive clinical efficacy remains unconfirmed.

## 1 Introduction

Based on the International Agency for Research on Cancer (IARC) data in 2022, approximately 20 million new cancer cases were diagnosed worldwide, resulting in about 9.7 million cancer-related deaths (1, 2). Metastasis remains the leading cause of cancer-related deaths (3). Common tumors such as lung cancer, breast cancer, ovarian cancer, and gastric cancer often show intracavitary dissemination and metastasis within serous cavities (pleural, peritoneal, and pericardial cavities), with a high incidence rate and the formation of malignant serous effusions (MSE) (4–6). MSE refers to the pathological accumulation of fluid in physiological serous cavities caused by the invasion of malignant tumor cells. It is not an independent disease but a common and serious complication of advanced cancer. MSE formation primarily results from direct tumor infiltration of serous membranes, leading to increased vascular permeability, lymphatic drainage obstruction, and excessive fluid production mediated by tumor-secreted inflammatory factors—biological mechanisms that render conventional therapies (drainage, chemotherapy) ineffective for durable control, as they fail to target the immunosuppressive tumor microenvironment and tumor heterogeneity driving effusion recurrence. Clinical manifestations correlate closely with the affected cavity: pleural effusion causes dyspnea, chest pain, and cough; ascites (peritoneal effusion) leads to abdominal distension, pain, and anorexia; pericardial effusion may cause cardiac compression, resulting in dyspnea and hemodynamic instability. The presence of MSE indicates poor prognosis and significantly diminished quality of life (QoL), underscoring the need for targeted immunotherapies that address these unique biological barriers. These effusions are associated with poor survival and prognosis. The development of malignant effusions related to serosal spread and metastasis further encourages widespread tumor implantation and spread throughout the body, including the pleura and peritoneum, and is linked to resistance and recurrence in late-stage treatment (7). Simultaneously, severe complications like dyspnea, chest pain, abdominal distension, and pain occur, seriously impacting patients’ quality of life (4, 8). Among all cancer patients, about 15% develop pleural effusion, and in certain cancer populations, approximately 54% present with ascites at initial diagnosis, representing a substantial patient cohort (4, 9). Notably, these effusions require pathological confirmation to distinguish malignant from non-malignant causes. However, there is a critical lack of universally effective treatments for malignant serous effusions due to metastasis to adequately reduce patient suffering, slow disease progression, and improve quality of life. Standard approaches like intracavitary chemotherapy often yield limited efficacy and cause significant toxicities. Faced with the constraints of conventional radiotherapy and chemotherapy, clinical researchers are increasingly focusing on immunotherapy—including immune checkpoint inhibitors, adoptive cell therapies, and oncolytic viruses—aiming to develop novel strategies targeting the immunosuppressive microenvironment of malignant effusions. This narrative review aims to critically synthesize the recent advances in immunotherapy strategies for malignant serous effusions (MSE), particularly focusing on malignant pleural effusion (MPE) and malignant ascites (MA). We will examine the emerging evidence from preclinical models and clinical trials for various immunotherapeutic approaches, including immune checkpoint inhibitors (ICIs), dendritic cell (DC) vaccines, oncolytic viruses, cytokines, monoclonal antibodies, and chimeric antigen receptor T-cell (CAR-T) therapy applied locally or systemically. The primary objective is to provide a comprehensive overview of the current landscape, evaluate the potential efficacy and limitations of these novel therapies, and discuss their implications for improving outcomes in patients with MSE.

## 2 Materials and methods (literature search strategy)

While this is a narrative review, not adhering to a full systematic review protocol, key literature was identified through searches on PubMed and Web of Science core collection databases. The primary search terms included combinations of: “malignant effusion” OR “malignant pleural effusion” OR “malignant ascites” OR “pericardial effusion” AND “immunotherapy” OR “immune checkpoint inhibitor” OR “CAR-T” OR “oncolytic virus” OR “dendritic cell vaccine” OR “cytokine”. Searches focused on articles published primarily within the last decade (2014–2024), encompassing preclinical studies, clinical trials (Phase I-III), case reports, and relevant reviews. Selection was based on the authors’ assessment of relevance, with priority given to clinical relevance (e.g., patient outcomes) and mechanistic novelty. Given the breadth of the topic and the narrative nature of this review, inclusion criteria were purposefully broad, seeking to capture the current state of advancement. Pericardial effusion is less emphasized due to its lower incidence and limited available immunotherapy research compared to pleural and peritoneal effusions.

Several biases may affect this review. Publication bias is a primary concern, as the preferential publication of positive findings could overstate the evidence. Secondly, the inclusion of diverse study designs—from randomized controlled trials to case-control and uncontrolled studies—reflects the limited availability of rigorous literature in this developing field. These non-randomized designs carry inherent risks, such as confounding and baseline imbalance, potentially reducing the robustness of our findings.

## 3 Existing clinical treatment options for malignant serous effusions

### 3.1 Treatment options for MPE

In recent years, some advancements have been made in treating MPE, but current approaches mainly focus on drainage methods. Presently, treatment is generally palliative (10), aimed at alleviating symptoms and enhancing QoL (11). Standard therapies fail to achieve durable control because they do not target the underlying immunosuppressive tumor microenvironment, persistent tumor cell seeding in the pleural cavity, or tumor heterogeneity—key biological barriers that drive effusion recurrence. The primary clinical option is thoracentesis, which can result in a cycle of protein loss and electrolyte imbalance (12). Over half of the patients need multiple procedures, making thoracentesis unsuitable as a long-term solution (13). Other treatment options for MPE include indwelling pleural catheters (IPC), pleurodesis with talc, pleurectomy, and pleuroperitoneal shunting (14). Currently, the role of chemotherapy in treating MPE remains debated, as there is limited evidence to confirm that systemic anticancer therapies can effectively improve MPE or that such treatments are safe and effective (13, 15, 16). Some studies (17–19) have explored intrapleural infusion of bevacizumab (a monoclonal antibody targeting vascular endothelial growth factor [VEGF]) and paclitaxel in patients with MPE caused by advanced non-small cell lung cancer (NSCLC). These approaches demonstrated efficacy comparable to chemotherapy alone, with a lower incidence and severity of toxic side effects. Recently, Guo et al. reported that in a phase I clinical trial, intrapleural infusion of tumor cell-derived microparticles encapsulating methotrexate significantly reduced MPE recurrence and improved patients’ symptoms and quality of life without notable toxic side effects (20). While these findings, including data from early-phase clinical trials and preclinical studies, offer promising ideas for MPE treatment, their safety and efficacy still require further validation in larger-scale studies. Although many researchers have proposed various strategies to enhance outcomes, finding truly effective methods remains challenging at present.

### 3.2 Treatment options for MA

Managing MA is challenging due to severe symptoms needing regular intervention, but there are no standardized guidelines, and most treatments lack high-quality trial support (21). Standard symptomatic and anti-tumor strategies often fail to provide durable control, as they cannot overcome peritoneal tumor seeding, immunosuppressive microenvironment, or tumor heterogeneity. Current treatment options for MA include both symptomatic and anti-tumor strategies. Symptomatic treatment mainly involves paracentesis, which effectively relieves symptoms like pain, bloating, and nausea, but does not prevent MA from recurring. Anti-tumor treatment is categorized into systemic and local approaches. According to retrospective data from Julia M. Berger et al., compared with best supportive care (BSC) alone, continuing systemic treatment after the diagnosis of MA is linked to improved survival and higher ascites response rates in a cohort of pancreatic cancer patients, although the potential side effects and impact on patients’ quality of life require further evaluation (22). Patients undergoing cytoreductive surgery combined with hyperthermic intraperitoneal chemotherapy (HIPEC) can greatly enhance overall survival (23), but about 80% face intraperitoneal recurrence within three years, as this approach cannot fully eliminate tumor cells in the peritoneal cavity (24–26).

In recent years, many attempts have been made to treat MA with different drugs. For example, losartan, which is approved by the U.S. Food and Drug Administration (FDA) for treating hypertension, has been studied for MA treatment (27). A phase II clinical trial showed that aflibercept, which targets VEGFA, VEGFB, and placental growth factor PIGF, can effectively prevent MA recurrence, but a major complication of this therapy is fatal gastrointestinal perforation (in 3 out of 29 patients) (28). Another phase II clinical trial showed that intraperitoneal bevacizumab can slow the reaccumulation of malignant ascites in women with chemotherapy-resistant epithelial ovarian cancer who have discontinued chemotherapy (29). Additionally, the matrix metalloproteinases (MMP) inhibitor Batimatstat has been proven to reduce tumor metastasis and malignant ascites production in animal studies. In ovarian cancer patients, intraperitoneal injection of Batimatstat has also decreased ascites volume in some cases. In a study among nine patients, five experienced ascites reduction, and the main adverse event was mild to moderate abdominal pain (30, 31). Furthermore, to provide a comprehensive overview of various malignant serous effusions (MSE) types and their associated treatments, we summarize key options in **Table 1**.

**Table 1:** Overview of malignant serous effusion types and associated clinical treatment options.

Malignant serous effusions (MSE)	Type description	Standard-of-care treatments	Investigational therapies	Key references
Malignant Pleural Effusion (MPE)	Common in lung, breast cancer; symptoms include dyspnea and pain. Often associated with poor prognosis and resistance to conventional therapies.	Thoracentesis, indwelling pleural catheter (IPC), talc pleurodesis, systemic or intrapleural chemotherapy.	Emerging immunotherapies such as ICIs (e.g., Sintilimab) and DC cell vaccines.	(10–12, 14, 15, 32, 33)
Malignant Ascites (MA)	Common in ovarian, gastric cancer; symptoms include abdominal distension and nausea. Frequently linked to peritoneal metastasis and high recurrence rates.	Paracentesis, cytoreductive surgery combined with hyperthermic intraperitoneal chemotherapy (HIPEC).	Targeted therapies (e.g., Batimatstat, Bevacizumab, Aflibercept), and immunotherapies including monoclonal antibodies (e.g., Catumaxomab) and CAR-T cells.	(22–24, 28–31, 34, 35)
Other Types	Includes pericardial effusion; less commonly discussed in clinical studies due to lower incidence.	Similar to MPE and MA, often involves local drainage	Immunosuppressive agents; immunotherapy options like oncolytic viruses are under investigation.	(4, 5, 8)

## 4 Advances in immunotherapy for malignant serous effusions

### 4.1 ICI therapy

Among ICIs, drugs targeting PD-1 and CTLA-4 are the most commonly used clinically. PD-1 inhibitors (e.g., nivolumab, pembrolizumab) restore T-cell cytotoxicity by blocking the interaction between PD-1 and its ligands PD-L1/PD-L2, while CTLA-4 inhibitors (e.g., ipilimumab) enhance T-cell activation and proliferation by preventing CTLA-4 from binding to B7 molecules. Both classes potentiate systemic anti-tumor immunity and have demonstrated significant efficacy across multiple solid tumors, offering a novel immunotherapeutic strategy for MSE (36, 37). Current research shows that tumor cells can evade immune cell antibody-dependent cellular cytotoxicity (ADCC) by overexpressing PD-L1, and activated naive B cells can inhibit Th17 cell proliferation through the PD-1/PD-L1 pathway, providing a scientific basis for PD-1 targeted therapy (34–36, 38–40). Preclinical studies have also offered valuable insights into the use of ICIs in treating malignant serous effusions. Kim et al. discovered in an MC38 colon cancer ascites model that STING (stimulator of interferon genes) agonists combined with anti-PD-1 can significantly improve peritoneal tumor blood vessel function and boost anti-cancer immunity (41). Dawen Zhao’s team found that in a mouse Lewis lung cancer MPE model, injecting liposomal nanoparticles with cyclic dinucleotides (LNP-CDN) directly into the pleural cavity activates the STING signaling pathway. This treatment enhances the immune response of the tumors, making them less ‘cold,’ and improves the effectiveness of tumor immunotherapy. Their results indicated that combining LNP-CDN with PD-L1 inhibitors decreased MPE production and pleural tumor burden more than administration of LNP-CDN alone, leading to longer survival in the MPE mice (42).

The integration of ICIs into first-line treatment for cancers commonly associated with effusions, such as non-small cell lung cancer (NSCLC), has shown tangible benefits. Although, a retrospective study in 2019 showed that the presence of MPE is an independent negative predictor of efficacy and survival for patients with non-small cell lung cancer (NSCLC) receiving anti-PD-1 monotherapy, as the presence of MPE in patients administered an anti‐PD‐1 antibody is associated with shorter PFS and OS (median PFS: 3.0 vs. 5.8 months, median OS: 7.9 vs. 15.8 months), regardless of the presence of PD‐L1 expression ≥ 1% of tumor cells (43). The same with a study in 2020, put evidence that MPE was independent predictorhistorical nihilismn patients treated with pembrolizumab (hazard ratio: 1.52, 95.0% confidence interval: 1.01-2.29; P = 0.043) (44). But a multicenter retrospective study in 2022 demonstrated that, for patients with nonsquamous NSCLC and MPE, first-line treatment with ICIs plus chemotherapy significantly improves PFS, OS, and response rates compared to immunotherapy alone (45). Furthermore, a key retrospective multicenter study in 2024 demonstrated that combination ICIs with chemotherapy significantly improved PFS in NSCLC patients with MPE compared to chemotherapy alone (median PFS: 7.4 vs. 5.7 months). Although this combination did not show a statistically significant improvement in median OS in this study, it established a new standard of care with a manageable safety profile (46). Otherwise, an exploratory clinical study indicated that seven patients who received intrapleural sintilimab after sufficient drainage exhibited an ORR of 66.7% at week 10, along with improved CD8+ T cell function (32). These evidence told us that even though MPE was a negative predictor of treatment efficacy, underscoring the presence of an MPE should not preclude patients from receiving effective systemic immunotherapy, moving away from historical nihilism.

In MA treatment, ICI therapy encounters notable hurdles. Fucà et al. examined 502 cases of mismatch repair-deficient (dMMR)/microsatellite instability-high (MSI-H) metastatic colorectal cancer and found that patients with ascites and peritoneal metastasis gained limited survival benefits from anti-PD-1 monotherapy. However, combining immunotherapies (anti-CTLA-4 and anti-PD-1) markedly increased overall survival (OS), underscoring the significance of combination approaches (47).

### 4.2 DC cell vaccines

Therapeutic cancer vaccines represent an immunotherapy approach, with dendritic cell (DC) vaccines being the most widely used in treating malignant serous effusions, though they have not translated into routine clinical use due to three critical barriers: high manufacturing complexity and costs, tumor antigen heterogeneity limiting universal targeting, and a lack of randomized controlled trials to confirm efficacy—limitations that must be emphasized to avoid overstating therapeutic promise. As essential antigen-presenting cells in the immune system, DCs have become a key target for immunotherapy of MSE (33). Multiple clinical studies have verified the safety and initial effectiveness of DC-based vaccine therapy. One study demonstrated that autologous DC vaccines, cultured from monocytes derived from MPE, were well tolerated (with no grade II/III toxicity) in eight patients with advanced lung cancer and successfully triggered tumor antigen-specific T cell responses in six patients (33). Takashi Morisaki and colleagues’ study supports this by using monocyte-derived DCs with activated lymphocytes and OK-432 (a streptococcal preparation) to treat five patients with chemotherapy-resistant MPE/MA. All patients showed a decrease in serous effusions and had a median survival time of over nine months (48). Additionally, in a human study involving 22 patients, Ai et al. developed an intraperitoneal (IP) DC-CIK therapy that offers multiple benefits: it significantly expands CD3+CD56+ CIK cells, decreases regulatory T cells (Tregs), and inhibits tumor growth and metastasis by promoting interferon-γ (IFN-γ) production. Their outcome analysis revealed 2 complete responses (CR), 7 partial responses (PR), and an ORR of 40.9% (49–52). Notably, DC-based vaccines have not translated into routine clinical use, primarily due to high manufacturing complexity and costs, tumor antigen heterogeneity that limits universal targeting, and substantial methodological variation across studies coupled with a lack of comparative controlled trials (51, 52). These limitations prevent definitive establishment of their efficacy and optimal use, emphasizing the need for standardized protocols and comparative studies.

### 4.3 Oncolytic viruses

Oncolytic virus therapy is an emerging approach for tumor treatment that selectively infects and destroys malignant cells (53), while also stimulating anti-tumor immune responses (54). It has demonstrated unique benefits in treating MSE. A phase I/IIa clinical trial involving intrapleural injections of oncolytic herpes simplex virus 1716 (HSV-1716) was conducted in 13 patients with malignant pleural mesothelioma (MPM). Clinical outcomes were evaluable in 12 patients, as one patient withdrew early following catheter fracture. The results confirmed that the treatment was safe and capable of inducing specific anti-tumor immune responses (55). A phase I clinical trial (NCT01766739) demonstrated that intrapleural injection of oncolytic vaccinia virus in patients with MPE elicited a local immune response. This response included increased levels of CD8+ T cells, NK cells, Tregs, DCs, and neutrophils in the pleural effusion post-treatment, along with a reduction in tumor cells and no serious adverse effects (56). Additionally, Alkayyal et al. observed in a CT26 colon cancer peritoneal metastasis model that IP injection of autologous tumor cells infected with oncolytic Maraba MG1 virus expressing IL12 *in vitro* enhanced the migration of activated NK cells to the peritoneal cavity, leading to a decreased tumor burden in tumor-bearing mice (57). A therapy using reovirus demonstrated in preclinical studies its ability to inhibit tumors by promoting the growth of CD3+ and CD8+ T cells and stimulating IFN secretion (58). Ulrich M Lauer and colleagues observed that oncolytic vaccinia virus GL-ONC1 reduced EpCAM-positive tumor cells in the ascites of patients with advanced peritoneal cancer (59). Zhou and team found that IP injection of oncolytic herpes simplex virus type 2 (OH2) in CT26 tumor-bearing mice induced a strong release of pro-inflammatory cytokines, mainly IL-6, along with increased infiltration of CD4+ and CD8+ T cells, leading to the elimination of ascites and significantly extending the survival of mice with CT26 tumors (60).

Existing evidence shows that oncolytic virus therapy has some effectiveness in treating MSE, but its clinical application continues to encounter obstacles like viral delivery efficiency and immunosuppression microenvironment.

### 4.4 Cytokines

Cytokines are widely used in a variety of benign and malignant diseases (1, 61–63). Recently, cytokine-based immunotherapy has gained recognition for its ability to modulate the immune system in the treatment of MSE.

IL-2 is a cytokine that promotes the activation, proliferation, and movement of T cells and NK cells (64). When administered locally, it can turn non-inflammatory tumors into inflammatory ones, which may boost the immune response to the tumor (65). Early research showed its promise as a standalone treatment: in 1993, a French team carried out the first phase I trial of intrapleural recombinant IL-2 for MPE (n=22), showing one CR, nine partial responses (PR), and an ORR of 45.5% (10/22), with manageable toxicity (59, 66, 67). Hu et al. conducted mechanistic studies showing that intrapleural injection of human recombinant IL-2 in patients with malignant pleural effusion (MPE) decreases PD-1 expression on pleural CD8+ T cells while increasing granzyme B and IFN-γ levels. This reverses T cell exhaustion and reduces carcinoembryonic antigen (CEA) levels (67). A meta-analysis supported combining IL-2: intrapleural IL-2 with cisplatin in MPE patients, significantly improving the ORR, disease control rate (DCR), and QoL compared to cisplatin alone (68). IL-2 has been widely used in MPE treatments. A retrospective study confirmed that intrapleural IL-2 and dexamethasone in children with pleural, peritoneal, or pericardial effusions from solid tumors or lymphoma can control effusions and boost 5-year overall survival (OS) (69).

The interferon (IFN) family includes subtypes such as IFN-α and IFN-β, which have attracted attention in treating MPE through their dual roles of activating the immune system and directly inhibiting tumor cell growth (70). These studies are historically relevant but not reflective of current clinical practice. In 1993, Goldman’s team conducted a landmark study that first showed the effectiveness of intrapleural IFN-α2b. The study involved 20 MPE patients, with a median response duration of 6 months and an ORR of 70% (14/20), including 8 CR and 6 PR, indicating significant efficacy (71). However, a prospective study in 2004 challenged this optimistic view: compared to standard bleomycin chemotherapy, patients receiving IFN-α2b had lower response rates and shorter survival (72). Additionally, both IL-2 and IFN-α2b are associated with significant toxicity (e.g., flu-like syndrome, capillary leak syndrome for IL-2) that limit tolerability, contributing to their declining clinical use in recent years.

### 4.5 Monoclonal antibodies

Monoclonal antibodies (mAbs) are immunoglobulins produced by identical immune cells derived from a single B-cell clone. Characterized by high specificity, extended serum half-life, and strong antigen-binding affinity, mAbs can selectively bind to target antigens on cells to mediate therapeutic effects such as immune cell recruitment for tumor lysis or signal blockade. They are widely applied in oncology, autoimmune disorders, and infectious disease management (73–75).

Catumaxomab is a rat-mouse bispecific trifunctional antibody that targets EpCAM (73, 74, 76). It promotes immune-mediated tumor destruction by binding to Fcγ receptors (types I, IIa, III) on EpCAM-positive tumor cells and immune cells such as NK cells and CD3+ T cells (77–79). In 2009, catumaxomab became the first drug approved in Europe for treating MA related to PC (41) (39), but it has since been withdrawn from the market due to commercial reasons and limited long-term efficacy data. Safety concerns include cytokine release syndrome and infusion-related reactions, which were observed in 39 grade 3 and 2 grade 4 treatment-related adverse events in a 16-patient study (34).

A trial involving 23 patients with chemotherapy-resistant ovarian cancer demonstrated that catumaxomab could prevent ascites buildup and effectively destroy tumor cells (34). A study by Heiss et al. confirmed that, compared to simple puncture treatment, combining catumaxomab with puncture significantly improved patients’ quality of life with EpCAM-positive MA, especially in managing pain and bloating, and the puncture-free survival time in the treatment group was notably longer (median 46 days vs. 14 days in the control group) (80). Emerging bispecific antibodies (e.g., M701 targeting EpCAM and CD3) are under investigation for MPE, but unresolved safety risks—particularly severe cytokine release syndrome—remain critical considerations for clinical development.

Regarding the safety of catumaxomab, in a study involving 16 patients, a total of 39 grade 3 and 2 grade 4 treatment-related adverse events (AEs) were observed, most of which were reversible and had no lasting effects. Overall, the safety was manageable (34). For the immunotherapy of MPE, a clinical trial NCT05543330 on M701 (a bispecific antibody targeting EpCAM and CD3) for treating malignant pleural effusion caused by NSCLC is currently underway.

Monoclonal antibodies, exemplified by the EpCAM-targeting catumaxomab, demonstrate clinical utility in alleviating MSE. However, broader application requires: (i) development of antibodies against novel tumor-associated antigens, (ii) optimization of dosing regimens to mitigate cytokine-release syndromes, and (iii) exploration of synergistic combinations with immunomodulators to enhance efficacy (80).

### 4.6 CAR-T cell therapy

#### 4.6.1 Basic principles of CAR-T therapy

**Figure 1:**
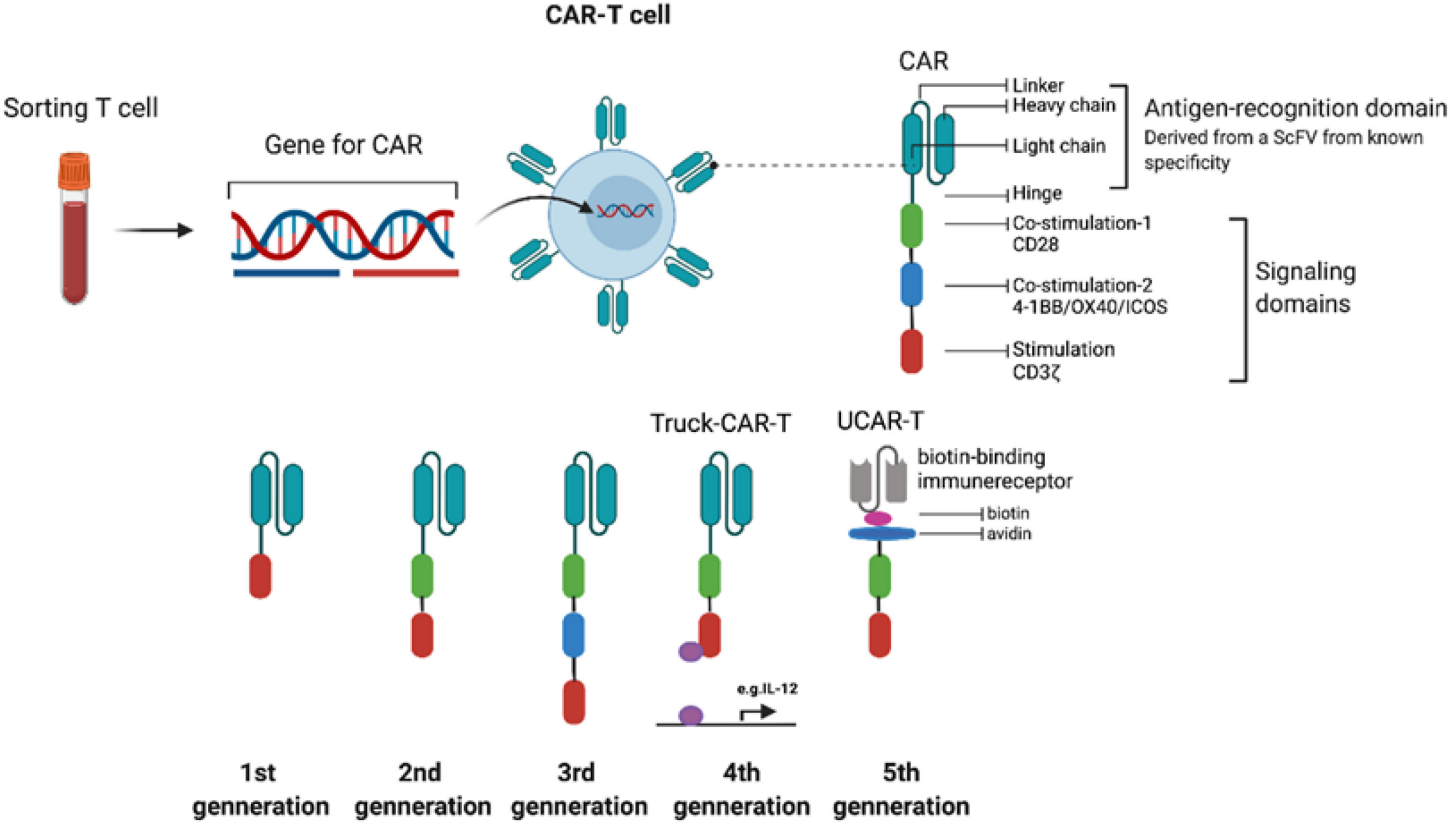
Schematic representation of CAR-T cell design and evolution. The chimeric antigen receptor (CAR) comprises an extracellular antigen-binding domain (derived from the scFv of a monoclonal antibody), a hinge, a transmembrane domain, and intracellular signaling domains responsible for T-cell activation. The lower panel illustrates the progressive evolution from first- to fifth-generation CAR-T cells, incorporating co-stimulatory and cytokine-secreting elements to enhance antitumor efficacy and persistence.

CAR-T cells are T cells engineered using genetic technology to express CAR molecules. They are characterized by MHC-independent activation and antigen-dependent targeted killing of tumor cells. The structure of a CAR, as shown in **Figure 1**, includes an antigen-binding domain, hinge, transmembrane domain, and intracellular signaling domains. The antigen-binding domain is the extracellular part of the CAR, functioning as the “GPS” locator for CAR-T cells, and is derived from the single-chain variable fragment (scFv) of a monoclonal antibody (81–83). Typically, the scFv is made up of the variable heavy chain (VH) and variable light chain (VL) from a monoclonal antibody, connected by a linker. The linker, a flexible connecting peptide commonly made of glycine and tryptophan, offers necessary flexibility and solubility, enabling VH and VL domains to fold properly and bind to antigens. Intracellular signaling domains typically consist of the ITAM region from the T cell receptor CD3ζ chain, along with co-stimulatory molecules such as CD28, 4-1BB (also called CD137 and TNFRSF9), OX40, or ICOS (inducible T cell costimulatory).

CAR-T technology has experienced five generations of modifications. All currently approved products are second-generation CAR-T cells, featuring intracellular costimulatory signaling molecules like CD28 and 4-1BB. The third, fourth, and fifth generations are still in clinical trials. The structure of CAR can be improved through genetic modifications of each functional component to boost CAR-T cell performance or reduce toxicity. For instance, the scFv can be optimized by screening for VHH.scFv from llama antibodies or by expressing “armored” proteins that allow T cells to autonomously produce or secrete immunomodulatory molecules such as IL-12, thereby enhancing immune activity and favorably altering the tumor microenvironment. Even TCR-deficient allogeneic universal CAR-T cells can be produced through TALEN or CRISPR-Cas9 gene-editing methods. These techniques lower costs, shorten manufacturing times, and enhance treatment universality by enabling recognition of a broader range of target antigens (84).

#### 4.6.2 Research progress of CAR-T cells in the treatment of tumor serosal metastasis

Research on CAR-T cell therapy for tumor serosal metastasis is still in early stages, with some phase I trials underway. Key real-world limitations include high manufacturing costs, limited scalability for widespread access, tumor antigen heterogeneity that may lead to treatment resistance, and toxicity risks (e.g., cytokine release syndrome, neurotoxicity) specific to intracavitary delivery—factors that must be balanced against preclinical and early clinical promise. Zhang Yan and colleagues reported using CD19-targeted CAR-T cells to treat a patient with relapsed follicular lymphoma and extensive chylous ascites. The CAR-T cells expanded within the ascitic fluid for three months, resulting in notable therapeutic benefits (85)—a finding that requires validation in larger cohorts. Oladapo O Yeku and colleagues developed an armored CAR-T cell that constantly secretes IL-12. *In vitro* tests revealed that these CAR-T cells showed strong proliferation and cytotoxicity even in the presence of immunosuppressive ascites. In a mouse model with intraperitoneal ID8-Muc16ecto tumors, the CAR-T cells maintained ongoing growth and demonstrated effective antitumor activity, which led to increased median survival and higher survival rates in a model of ovarian cancer with peritoneal metastasis (35). John P Murad and colleagues developed a humanized TAG72-specific CAR with a 4-1BB intracellular costimulatory signaling domain (TAG72-BBζ). *In vitro* studies demonstrated that TAG72-BBζ CAR T cells effectively killed various TAG72-positive ovarian cancer cell lines and proliferated and killed efficiently in patient-derived ovarian cancer ascites. In animal tests, intraperitoneal injection of TAG72-BBζ CAR T cells significantly slowed tumor growth, extended the overall survival of mice, and further improved results with repeated CAR T cell infusions (86). Cansu E Önder and team validated the effectiveness of AdCAR-T cells and biotinylated monoclonal antibodies in a 3D organoid model from four patients with malignant pleural effusion, confirming AdCAR-T’s activity against metastatic breast cancer (87).

## 5 Discussion

The immunotherapy landscape for malignant serous effusions (MSE) is rapidly evolving, presenting promising alternatives to traditional palliative approaches burdened by limited efficacy and significant toxicities. This review synthesizes evidence supporting the potential of diverse immunotherapeutic modalities—including immune checkpoint inhibitors (ICIs), CAR-T cells, oncolytic viruses, dendritic cell (DC) vaccines, cytokines (e.g., IL-2), and targeted antibodies (e.g., catumaxomab)—delivered either locally or systemically. However, a critical appraisal reveals that the current evidence base, while encouraging, is predominantly derived from early-phase clinical trials (largely Phase I/II) and preclinical models. Many cited studies (32, 34, 35, 55, 56, 60, 66, 68, 80, 86) are constrained by limitations common to exploratory research: small sample sizes, non-randomized designs, single-arm cohorts, potential selection bias, and heterogeneity in patient populations, disease stages, and prior treatments. For instance, the promising ORR of 66.7% reported for intrapleural sintilimab was observed in merely 7 patients (32), while catumaxomab efficacy was demonstrated in studies with limited cohorts (34, 80). Although CAR-T studies exhibit remarkable biological activity in ascites models (35, 86), translating this into robust clinical efficacy within larger, heterogenous patient groups remains a significant challenge. A critical comparison of immunotherapeutic modalities reveals distinct profiles in terms of clinical readiness, safety, feasibility, and durability of response: (1) ICIs have the highest clinical readiness but larger-scale clinical trials are still needed to validate their efficacy; (2) oncolytic viruses and DC vaccines are feasible but lack randomized data on long-term durability; (3) CAR-T cells exhibit strong preclinical activity but are limited by manufacturing costs, scalability, and toxicity risks; (4) cytokines have historical response data but significant toxicity concerns. Local delivery strategies—such as intracavitary administration of ICIs, cytokines, oncolytic viruses, and CAR-T cells—aim to maximize exposure within the effusion microenvironment while potentially minimizing systemic toxicity, as suggested by favorable tolerability in several studies (32, 34, 55, 56, 66). Notably, intrapleural LNP-CDN combined with PD-L1 blockade demonstrated superior tumor control versus systemic therapy in a preclinical malignant pleural effusion (MPE) model (42), underscoring the advantage of targeting the local immunosuppressive niche. In contrast, systemic ICI therapy appears less effective in the presence of MPE (43, 44), potentially reflecting the unique, potent immunosuppression within the serous cavity that differs from the primary tumor site. Data analysis by Grosu et al. (88), however, suggests potential benefit if the primary tumor is responsive, warranting further investigation. The unique pathophysiology of MSE poses specific hurdles, characterized by a profoundly immunosuppressive tumor microenvironment (TME) within the effusion fluid. This milieu features high levels of immunosuppressive cells (Tregs, MDSCs), cytokines (TGF-β, IL-10), metabolic dysregulation (lactic acid, hypoxia), and physical barriers, impairing immune cell infiltration, activation, and function (41, 42, 49). Strategies like STING agonists (41, 42) or “armored” CAR-T cells engineered to secrete IL-12 (35) aim to counteract this suppression by converting immunologically “cold” TMEs into “hot,” inflamed ones. The high degree of tumor heterogeneity and clonal evolution within effusions further complicates the development of effective targeted immunotherapies. Consequently, while preclinical and early clinical results across these diverse immunotherapeutic avenues are undeniably promising, their definitive clinical utility necessitates rigorous validation through larger, randomized controlled trials specifically designed to address patient heterogeneity and directly compare the efficacy, safety, and mechanistic advantages of local versus systemic delivery strategies within this complex disease context.

## 6 Limitations and future perspectives

This review has several inherent limitations, largely reflecting the limitations of the current literature base itself. Primarily, the lack of large-scale, randomized controlled trials (RCTs) comparing novel immunotherapies against standard of care is an urgent research gap, as RCTs are needed to confirm clinical efficacy. Additional high-priority gaps include the absence of validated predictive biomarkers for patient stratification, limited data on combination strategies to overcome TME immunosuppression, and incomplete understanding of optimal delivery methods for intracavitary immunotherapies. The heterogeneity in study designs, patient populations, treatment protocols, and outcome measures across the cited literature complicates direct comparisons and meta-analyses.

Future research must prioritize:

- Conducting well-designed, adequately powered Phase II/III RCTs to establish the true clinical efficacy and safety profiles of these promising immunotherapies compared to current standards. Trials should incorporate patient-reported outcomes (PROs) and quality of life (QoL) measures as crucial endpoints alongside traditional efficacy metrics.
- Developing predictive biomarkers to identify patients most likely to benefit from specific immunotherapies. Deep characterization of the effusion TME (cellular composition, cytokine profile, genomic landscape, PD-L1 status) is essential for patient stratification.
- Optimizing combination strategies: Exploring rational combinations (e.g., ICI + oncolytic virus (41), CAR-T + ICIs, local + systemic therapies) holds significant promise for overcoming resistance mechanisms within the effusion TME.
- Improving delivery and engineering: Enhancing the delivery efficiency and persistence of locally administered agents [e.g., engineered oncolytic viruses with higher tumor selectivity, optimized CAR-T constructs for the effusion TME like armored CAR-T (35), improved nanoparticle formulations (42)] is critical. Research into universal or “off-the-shelf” cellular therapies could improve accessibility.
- Understanding and targeting the unique MSE TME: Continued mechanistic studies are vital to fully elucidate the immunosuppressive pathways operational within malignant effusions and identify novel targets for intervention.

## 7 Conclusion

In conclusion, immunotherapy represents a dynamic and rapidly advancing frontier in the management of malignant serous effusions, shifting the paradigm from purely palliative care towards potential disease-modifying strategies, though it remains investigational. Challenges include the immunosuppressive effusion microenvironment, tumor heterogeneity, and the current predominance of early-phase evidence—definitive clinical benefit has not yet been confirmed in large randomized trials. The reviewed approaches (ICIs, CAR-T cells, oncolytic viruses, DC vaccines, cytokines, and targeted antibodies) show preliminary potential, particularly with local delivery modalities in early trials, underscoring the importance of continued investigation. Realizing the full therapeutic promise of immunotherapy for MSE necessitates addressing the current limitations through rigorous large-scale clinical trials, biomarker development, optimization of combination regimens and delivery strategies, and a deeper mechanistic understanding of the effusion TME. Collaborative efforts between clinicians and researchers are paramount to translate these innovative approaches into tangible improvements in survival and quality of life for patients suffering from these challenging complications of advanced cancer.
